# Successful pacemaker implantation using left bundle branch area pacing in a patient with dextrocardia: A case report

**DOI:** 10.1002/joa3.70118

**Published:** 2025-06-19

**Authors:** Yoshiro Tsuruta, Toshihiko Goto, Yomei Sakurai, Kento Mori, Yoshihiro Seo

**Affiliations:** ^1^ Department of Cardiology Nagoya City University Graduate School of Medical Sciences Nagoya Japan

**Keywords:** complete atrioventricular block, dextrocardia, left bundle branch area pacing, pacemaker, stylet‐driven leads

## Abstract

The left image shows an intraoperative fluoroscopic view with left–right inversion, and the right image is a postoperative noncontrast CT. Both demonstrate the right ventricular lead positioned in the interventricular septum.

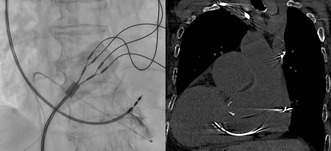

A 96‐year‐old woman was brought to our emergency room for dyspnea. A 12‐lead electrocardiogram showed complete atrioventricular block with a ventricular rate of 37 beats/min, negative QRS waves in leads I and aVL, and inverted P waves in leads I and aVL (Figure 1). A chest radiograph (Figure 2A) and a computed tomography scan (Figure 2B) showed that this patient had dextrocardia. The patient was receiving medication for hypertension but none that could induce bradycardia. No other secondary causes of bradycardia were identified. We diagnosed symptomatic bradycardia due to complete atrioventricular block and proceeded with pacemaker implantation.

**FIGURE 1:**
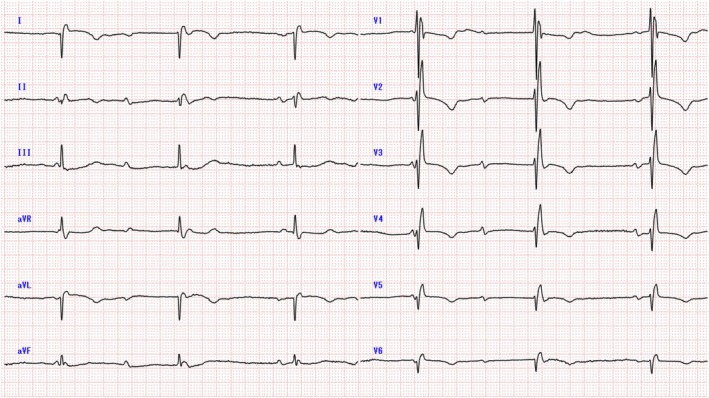
A 12‐lead electrocardiogram at presentation showing complete atrioventricular block with a ventricular rate of 37 beats/min, negative QRS waves in leads I and aVL, and inverted P waves in leads I and aVL.

**FIGURE 2:**
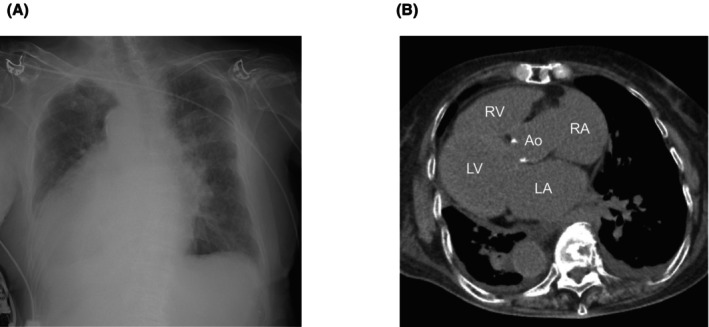
Both the chest radiograph (posteroanterior view) (A) and computed tomography images (B) confirmed that the patient had dextrocardia. Ao, aorta; LA, left atrium; LV, left ventricle; RA, right atrium; RV, right ventricle.

Given the patient's anatomical anomaly, we adapted our procedure by inverting the display settings of the angiographic system to account for the reversed left–right and anterior–posterior orientations. This adjustment allowed the operator to interpret the fluoroscopic images as if performing a standard procedure, minimizing potential confusion during lead placement. We also positioned the 12‐lead electrocardiogram symmetrically to resemble waveforms of patients with normal anatomy. A right‐sided transvenous approach was chosen because of dextrocardia. Left bundle branch area pacing (LBBAP) was selected to achieve more physiological ventricular contraction. Using a delivery sheath (Selectra 3D‐55‐39, BIOTRONIK, Berlin, Germany), a right ventricular pacing lead (Solia S 60, BIOTRONIK) was placed in the interventricular septum. When the delivery sheath was inserted directly, it initially pointed toward the right ventricular free wall. To adjust its direction, we extended the sheath's second curve while referring to fluoroscopic imaging. After repeating this maneuver several times, we successfully redirected the sheath toward the septum. Figure 3A is an intraoperative image taken at right anterior oblique 20° and mirrored to simulate a left anterior oblique 20° view. The right atrial lead (Solia S 45, BIOTRONIK) was placed in the right atrial appendage, and a dual‐chamber pacemaker (Amvia Sky DR‐T, BIOTRONIK) was set to 60 beats/min. Unlike ventricular lead placement, no special techniques were required to insert the atrial lead into the right atrial appendage. The total procedure time was 95 min. Figure 3B shows a postoperative image with left–right mirroring, and Figure 4A presents the electrocardiographic findings. Supplementary Figures S1 and S2 show postoperative noncontrast CT scans that confirm the position of the right ventricular lead. During the procedure, the intracardiac electrocardiogram showed a QRS complex duration of 121 ms, a left ventricular activation time in lead V6 of 76 ms, and a V6–V1 interpeak interval of 39 ms (Figure 4B). After implantation, her symptoms improved. At the 1‐week follow‐up, the ventricular pacing threshold was 0.7 V at 0.4 ms, with a ventricular pacing rate of 100%.

**FIGURE 3:**
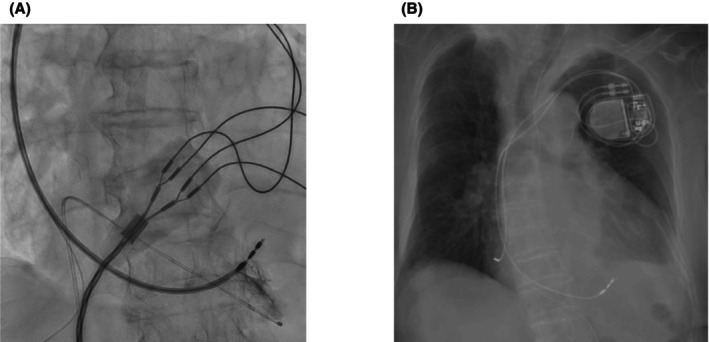
(A) shows an intraoperative image taken at right anterior oblique 20° and mirrored to simulate a left anterior oblique 20° view. During the procedure, imaging was performed with a setup in which the left–right orientations were reversed. Contrast material injection reveals the lead positioned deep in the interventricular septum in the left–right mirrored image. (B) shows a postoperative image with left–right mirroring.

**FIGURE 4:**
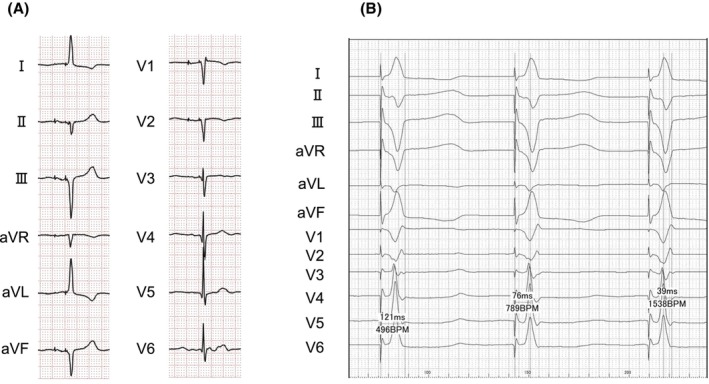
(A) A 12‐lead electrocardiogram after pacemaker implantation, showing reversed arm and leg leads, as well as right‐sided chest leads. (B) The intracardiac electrocardiogram showed a QRS complex duration of 121 ms, a left ventricular activation time of 76 ms in lead V6, and a V6–V1 interpeak interval of 39 ms.

LBBAP is gaining popularity as a physiological pacing strategy; however, its application in patients with dextrocardia presents unique challenges. In such cases, catheter manipulation is reversed, and commercially available devices are designed for standard anatomical configurations, increasing the complexity of implantation. Previous reports have described the manual inversion of delivery sheaths to address this issue.^1^ In our case as well, we needed to reshape the second curve of the guiding catheter several times to direct it toward the septum. We also utilized an angiographic device setting that effectively mirrored the left–right orientations. This approach allowed for a more intuitive procedural workflow, closely resembling standard pacemaker implantation. The primary advantage was improved spatial orientation for the operator, reducing potential confusion during lead placement. We would like to emphasize that in patients with dextrocardia, mirrored fluoroscopic imaging is particularly useful not only for LBBAP but also for other procedures requiring precise lead placement, such as coronary sinus lead positioning in CRT and low‐septal pacing. Additionally, our decision to implant the pacemaker in the right chest wall facilitated a smoother implantation process, further reducing procedural complexity. This case demonstrates that by optimizing visualization techniques, LBBAP can be successfully performed even in patients with dextrocardia.

A previous report described His bundle pacing using a lumenless lead in a patient with dextrocardia.^2^ In our case, we instead used a stylet‐driven lead, primarily because it is more commonly used at our hospital. Additionally, studies suggest that stylet‐driven leads result in shorter procedure times compared to lumenless leads.^3^ Therefore, stylet‐driven leads may be the preferable option for elderly patients with heart failure when minimizing procedure time is a priority.

## 1 CONCLUSION

This case highlights the technical feasibility of LBBAP in a patient with dextrocardia. The use of a mirrored angiographic display can help overcome anatomical challenges and facilitate successful LBBAP in such patients. Additionally, stylet‐driven leads, which are associated with shorter procedure times, may be the preferred option when procedural efficiency is a priority.

## FUNDING INFORMATION

Not Applicable.

## CONFLICT OF INTEREST STATEMENT

Authors declare no conflict of interests for this article.

## ETHICS STATEMENT

Ethical approval was not required as this is a case report.

## ETHICS AND INTEGRITY STATEMENT

This case report complies with the ethics and integrity policies of the *Journal of Arrhythmia*.

## PATIENT CONSENT STATEMENT

Written informed consent was obtained from the patient for publication of this case report and accompanying images.

## CLINICAL TRIAL REGISTRATION

Not Applicable.

## PERMISSION TO REPRODUCE MATERIAL

Not Applicable.

## Supporting information


Figure S1.

Figure S2.

Supinfo S1.

## Data Availability

Data sharing is not applicable to this article, as no datasets were generated or analyzed.

## References

[1] Bodagh N , Malaczynska‐Rajpold K , Eysenck W , O'Connor M , Wong T . Left bundle area pacing for tachycardia‐bradycardia syndrome in a patient with dextrocardia. JACC Case Rep. 2022;4:1213–1217. 36213881 10.1016/j.jaccas.2022.07.019 PMC9537106

[2] Yoshimoto D , Sakamoto Y , Yamaguchi R , Suzuki T . Permanent his‐bundle pacing for dextrocardia with situs inversus totalis using a combination of an electrode catheter and a deflectable sheath. HeartRhythm Case Rep. 2019;5:549–551. 31890571 10.1016/j.hrcr.2019.08.010 PMC6926253

[3] Cano Ó , Navarrete‐Navarro J , Zalavadia D , Jover P , Osca J , Bahadur R , et al. Acute performance of stylet driven leads for left bundle branch area pacing: a comparison with lumenless leads. Heart Rhythm O2. 2023;4:765–776. 10.1016/j.hroo.2023.11.014 PMC10774671 38204462

