# Supplementary figures and images for: Successful pacemaker implantation using left bundle branch area pacing in a patient with dextrocardia: A case report
Source: J Arrhythm. 2025 Jun 19;41(3):e70118. doi: 10.1002/joa3.70118 (PMC12177230; doi:10.1002/joa3.70118)

## Slide 1
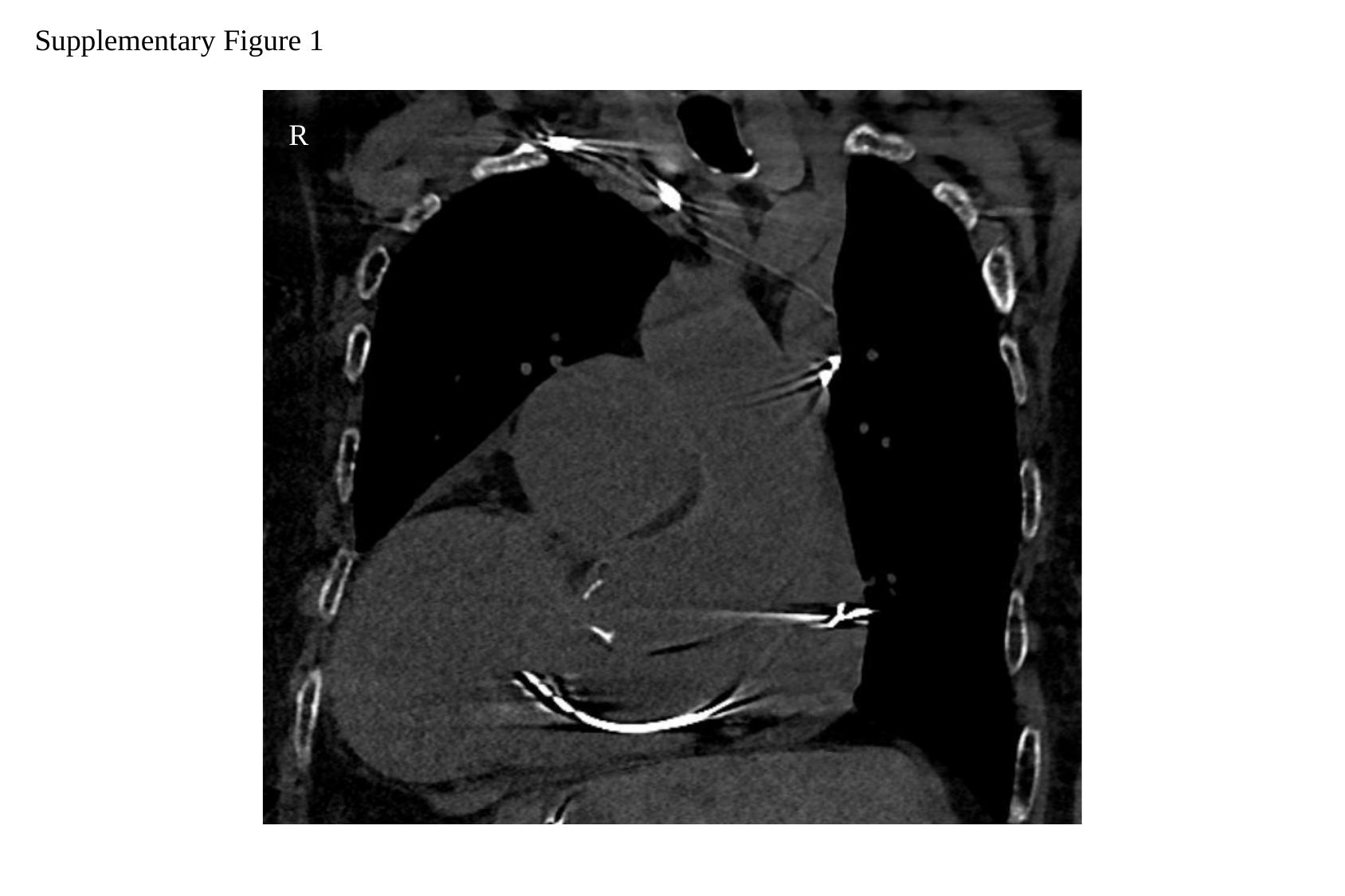

Supplementary Figure 1
R

Supplement: Supplementary file 1 — Figure S1. [file JOA3-41-e70118-s002.pptx]

## Slide 1
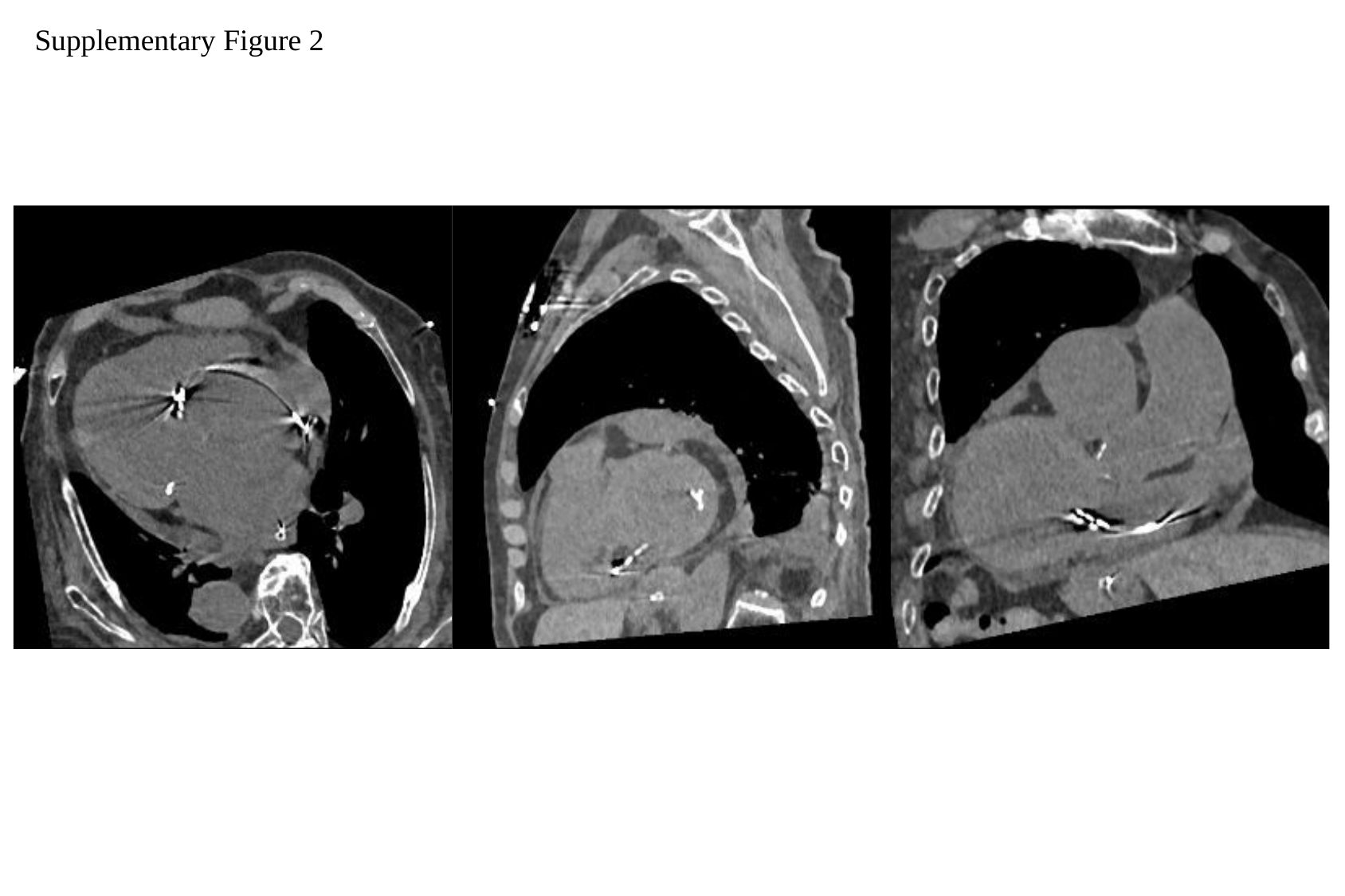

Supplementary Figure 2
R

Supplement: Supplementary file 2 — Figure S2. [file JOA3-41-e70118-s001.pptx]
