# Supplementary material for: Successful pacemaker implantation using left bundle branch area pacing in a patient with dextrocardia: A case report
Source: J Arrhythm. 2025 Jun 19;41(3):e70118. doi: 10.1002/joa3.70118 (PMC12177230; doi:10.1002/joa3.70118)
Supplement: Supplementary file 3 — Supinfo S1. [file JOA3-41-e70118-s003.docx]

**Supplementary material**

Supplementary Figure 1. A postoperative non-contrast CT scan showed the position of the right ventricular lead.

Supplementary Figure 2. Multi-planar reconstruction (MPR) images from three directions are presented. Although ECG-gated CT was used, image resolution near the lead tip was limited due to halation. Nevertheless, these MPR images provide useful information for understanding the orientation of the lead.
